# EfficientSegNet: Lightweight Semantic Segmentation with Multi-Scale Feature Fusion and Boundary Enhancement

**DOI:** 10.3390/s25195934

**Published:** 2025-09-23

**Authors:** Le Zhang, Mengwei Li, Peng Zhang, Peng Liu

**Affiliations:** 1 School of Instrument and Electronics, North University of China, Taiyuan 030051, China; b20230634@st.nuc.edu.cn (L.Z.); zhangpeng6@nuc.edu.cn (P.Z.); 2 School of Electrical and Control Engineering, North University of China, Taiyuan 030051, China; pengliu@nuc.edu.cn; 3 North Automatic Control Technology Institute, Taiyuan 030006, China

**Keywords:** semantic segmentation, deep learning, artificial intelligence, computer vision

## Abstract

Semantic segmentation is a crucial task in computer vision with broad applications in autonomous driving, intelligent surveillance, drone vision, and other fields. The current high-precision segmentation models generally suffer from large parameter sizes, high computational complexity, and substantial memory consumption, which limits their efficient deployment in embedded systems and resource-constrained environments. In addition, traditional methods exhibit significant limitations in handling multi-scale targets and object boundaries, particularly during deep feature extraction, where the loss of shallow spatial information often results in blurred boundaries and reduced segmentation accuracy. To address these challenges, we propose EfficientSegNet, a lightweight and efficient semantic segmentation network. This network features an innovative architecture that integrates the Cascade-Attention Dense Field (CADF) module and the Dynamic Weighting Feature Fusion (DWF) module, effectively reducing computational resource requirements while balancing global semantic information and local detail recovery. Experimental results demonstrate that EfficientSegNet achieves an excellent balance between segmentation accuracy and computational efficiency on multiple public datasets, providing robust support for real-time segmentation tasks and applications on resource-constrained devices.

## 1. Introduction

With the continuous evolution of artificial intelligence and the widespread application of deep learning technologies, semantic segmentation has become one of the most essential tasks in computer vision. It plays a vital role in numerous real-world applications, such as autonomous driving [1], medical image analysis [2], intelligent surveillance [3], and drone-based scene understanding [4]. Unlike coarse-grained classification or bounding-box detection, semantic segmentation assigns a category label to each individual pixel in an image, thereby enabling high-precision scene interpretation. This fine-grained recognition capability forms the foundation for higher-level visual reasoning tasks, including object detection [5], spatial localization [6], target tracking [7], and comprehensive environmental perception [8].

Despite decades of research, designing robust and efficient semantic segmentation algorithms remains challenging. Earlier algorithmic solutions—although efficient in specific scenarios—largely depend on manually crafted low-level image features, which inherently lack adaptability to high intra-class variation or spatial context dynamics. Such limitations become particularly evident when segmenting cluttered environments with densely distributed targets or ambiguous boundaries [9,10,11].

With the rapid development of deep learning, Convolutional Neural Networks (CNNs) have gradually become dominant in semantic segmentation research. By learning from large amounts of image data, CNNs can automatically extract high-level features, achieving more accurate and refined semantic segmentation. However, these methods inherently rely on local convolution operations, which limit their ability to model long-range dependencies and often result in insufficient capture of global contextual information. To address the challenges posed by the coexistence of multi-scale targets and complex spatial contexts, a series of multi-scale feature fusion modules have been proposed, such as Atrous Spatial Pyramid Pooling (ASPP) and its extended version DenseASPP [12]. These methods significantly expand the receptive field and enhance the model’s ability to represent multi-scale features by introducing dilated convolutions or densely connected structures with different dilation rates, thereby improving the modeling of semantic consistency and spatial structural details in complex scenes. However, dilated structures are always sparsely sampled, which not only dilutes the feature response related to the target but also introduces significant background noise, resulting in the loss of local detail information, and determining a reasonable combination of dilation rates is still a problem that needs further exploration. Meanwhile, this architecture typically relies on feature concatenation or cascading operations, which can sharply increase channel dimensions and introduce substantial redundant information. This not only raises computational costs but also weakens the representation of effective features.

In recent years, Transformer-based semantic segmentation models have achieved state-of-the-art performance on multiple benchmark datasets. Although these models demonstrate excellent capabilities in modeling long-range dependencies and integrating global context, their high computational and memory overhead makes them difficult to deploy on resource-constrained mobile or embedded platforms [13]. In contrast, convolutional neural network (CNN)-based models remain the mainstream choice for embedded systems and mobile devices due to their efficiency and hardware compatibility.

To address these issues, attention mechanisms have been introduced into CNN-based semantic segmentation networks. By adaptively assigning weights to features, these mechanisms highlight important spatial regions and channel information while preserving global contextual cues, thereby alleviating feature redundancy, improving model efficiency, and enhancing semantic prediction consistency. Zhang et al. [14] effectively integrated global and local information through a global local attention module and multi-scale feature fusion, significantly improving the accuracy and real-time performance of drone image segmentation. Lian et al. [15] proposed a lightweight semantic segmentation network, which achieved efficient segmentation through ghost dependent separable revolution with efficient channel attention block and lightweight feature pyramid network. Mo et al. [16] enhanced the adaptive weighting of key channel and spatial position semantic information by introducing a unit attention module, thereby improving the segmentation accuracy of non-structured road drivable areas. Li et al. [17] used multi-layer cross attention module (MCAM) and efficient channel attention module (ECAM) to enhance the interaction between position and channel features and combined semantic aggregation pyramid pooling (SAPPM) to achieve multi-scale feature fusion.

Although the attention mechanism reduces feature redundancy by emphasizing key features, it only reweights existing features and cannot fundamentally recover the spatial structure lost through multiple downsamplings. To mitigate this problem, existing methods typically incorporate shallow features to supplement the representation. However, even state-of-the-art approaches struggle to strike a balance between semantic abstraction and spatial precision. Specifically, deep feature representations (rich in semantics) often lose detailed spatial structures because of repeated downsampling. Conversely, shallow features preserve local spatial textures but lack the semantic depth necessary for accurate region-level classification. The simple merging of these features fails to fully utilize their complementary advantages, often resulting in fuzzy object boundaries, semantic ambiguity, or redundancy in feature maps.

To address these challenges, we propose EfficientSegNet, a novel lightweight semantic segmentation framework that emphasizes the efficient integration of multi-scale semantic abstraction and boundary-aware spatial refinement. The core architecture is designed to bridge the semantic gap between deep and shallow features while ensuring minimal computational overhead, thereby enabling real-time segmentation on mobile or embedded platforms. Through the integration of hierarchical receptive fields, attention-guided fusion, and dynamic weighting mechanisms, EfficientSegNet aims to overcome the limitations of existing approaches in handling redundant information and preserving object boundaries in complex scenes.

The main contributions of this work are as follows:

(1) Cascade Attention Dense Field (CADF) Module: The CADF module captures deep semantic information through progressively enlarged multi-scale receptive fields. Information sharing across scales is facilitated to maintain high-resolution representations and produce finer segmentation outputs.
(2) Dynamic Weighting Fusion (DWF) Module: The DWF module supervises the fusion process of deep and shallow features by dynamically weighting feature contributions. This mechanism compensates for resolution-induced information loss and enhances boundary clarity.
(3) Lightweight Architecture: EfficientSegNet employs a resource-efficient design that significantly reduces computational demands, enabling real-time inference on mobile or resource-constrained devices without compromising segmentation accuracy.

## 2. Related Work

The field of semantic segmentation has made significant progress, evolving from traditional image processing techniques to modern deep learning-based solutions, and more recently to Transformer-based architectures. Traditional methods, such as thresholding, edge detection, region growing, and graph-based approaches, operate on pixel-level intensity or texture features [18,19,20,21]. While computationally inexpensive, these techniques typically lack robustness in complex scenes, and their performance is highly sensitive to noise and scene variation.

With the rise of deep learning, convolutional neural networks (CNNs) have enabled end-to-end learning of semantic representations. Fully Convolutional Networks (FCN) were among the first to replace fully connected layers with convolutional layers, enabling dense pixel-wise predictions on images of arbitrary size [22]. This innovation laid the foundation for more advanced architectures such as U-Net, which introduced an encoder–decoder structure with skip connections to restore spatial detail [23]. Subsequent developments, including PSPNet [24] and HRNet [25], emphasized multi-scale contextual understanding and high-resolution feature preservation. Meanwhile, the DeepLab series integrated dilated convolutions and spatial pyramid pooling modules to enhance receptive field coverage and contextual aggregation [26,27,28,29], providing an important foundation for subsequent research on multi-scale feature fusion. Building on this, ConTriNet [30] proposed the Residual Atrous Spatial Pyramid Module (RASPM), which combines multi-branch atrous convolution with residual connections to aggregate multi-scale contextual information while preserving spatial details, thereby significantly improving feature representation capabilities. LiteFusionNet [31] designed Efficient Atrous Spatial Pyramid Pooling (EASPP), which reduces computational complexity by lowering the dilation rate and introduces additional upsampling operations to enhance the fusion of semantic and spatial information. SWDE Net [32] proposed Cross-Contextual Atrous Spatial Pyramid (CC-ASP), which adopts a cascaded structure to achieve stronger multi-scale contextual modeling. However, if dilated convolutions are stacked excessively or the dilation rate is improperly set, they not only struggle to cover key feature regions effectively but also introduce significant noise, leading to spatial structure blur and semantic information loss. In addition, multi-branch or cascaded structures often introduce substantial redundant computation, limiting inference efficiency.

To overcome the limitations of heavyweight models, researchers have focused on designing lightweight networks that reduce computational cost while preserving segmentation accuracy [33,34,35]. Depthwise separable convolutions, as utilized in the MobileNet family [36,37,38], significantly reduce the number of parameters and floating-point operations. ShuffleNet introduced channel shuffling and grouped convolutions to further enhance efficiency [39]. Meanwhile, some studies have introduced improved feature fusion modules or attention mechanisms to remove redundant features and highlight key semantics, thereby enhancing global semantic consistency while maintaining a lightweight design. SFRSeg [40] proposes a semantic-guided feature recombination strategy based on a lightweight backbone, achieving a better balance between accuracy and efficiency through multi-level feature fusion and attention enhancement. BiSeNet v3 [41] adopts a dual-branch design, effectively combining deep branches for extracting global contextual information with shallow branches for preserving local details via a feature fusion module. MRBANet [42] introduces a lightweight multi-resolution branch attention neural network that adaptively adjusts the fusion weights of each branch to highlight prominent contextual features. LMFDCNet [43] employs three sub-networks to extract multi-level features and utilizes a three-edge fusion module to enhance low-level details and high-level semantic features, thereby enabling more accurate object representation in complex environments. Although these methods achieve a good balance between accuracy and efficiency, they still face issues related to insufficient feature fusion or fixed fusion weights. The downsampling process is irreversible, and even with upsampling interpolation, the lost high-frequency texture and boundary information cannot be recovered. As a result, deep and shallow features become semantically and spatially inconsistent. Existing feature fusion methods mainly rely on concatenation or weighting strategies, lacking effective semantic alignment and spatial correction mechanisms, which limits the improvement of model performance in detail perception and boundary localization.

Recently, Transformer-based architectures have garnered attention for their capacity to capture global dependencies through self-attention mechanisms. SegFormer, for instance, introduces a hierarchical Transformer encoder tailored for segmentation tasks [44]. Swin Transformer enhances computational efficiency using a shifted window strategy and patch merging hierarchy [45], while Mask2Former integrates mask-based decoding with attention modules to improve segmentation quality [46]. Although these models achieve competitive performance, their practical deployment is hampered by high resource demands and dependency on large-scale annotated datasets [47,48,49].

Despite these diverse approaches, several key challenges remain unresolved: (1) Multiple downsampling in CNN backbone networks irreversibly discards high-frequency texture and boundary information, resulting in severe degradation of the spatial representation ability of deep features and making it difficult to accurately characterize small targets and fine-grained structures. (2) The fusion process of deep and shallow features lacks an adaptive adjustment mechanism, which makes it difficult to alleviate semantic and spatial misalignment. (3) Although multi-scale perception structures enhance feature representation, they often introduce a large amount of redundant computation and parameter overhead, limiting the practical deployment of models on resource-constrained platforms such as embedded or mobile devices.

To this end, our EfficientSegNet architecture is designed to unify deep semantic abstraction and shallow spatial cues through attention-guided modules and dynamic fusion strategies. By addressing both computational constraints and segmentation quality, the proposed model offers a practical and scalable solution for real-world semantic segmentation.

## 3. Methodology

### 3.1. Network Architecture

**Figure 1:**
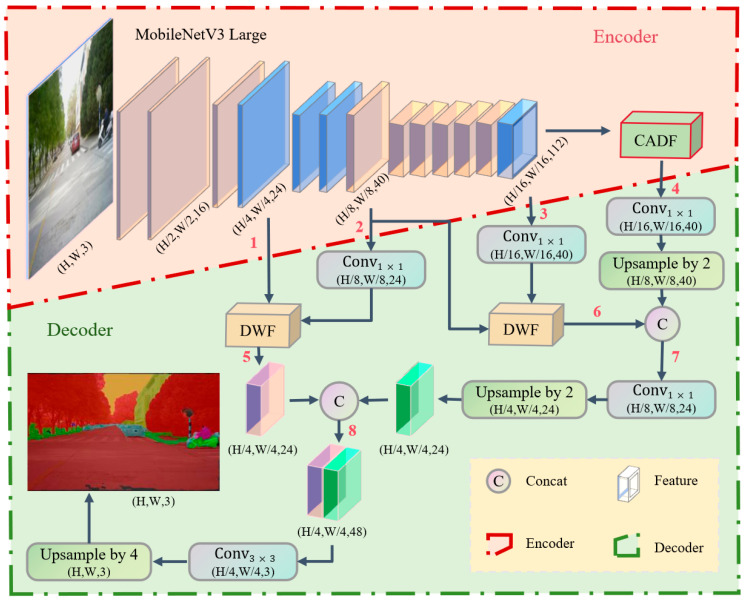
Schematic of EfficientSegNet network structure.

The overall architecture of the proposed EfficientSegNet is illustrated in Figure 1. The network enhances semantic representation and segmentation accuracy while maintaining computational efficiency. It consists of lightweight backbone network, CADF Module, and DWF Module.

In the feature extraction phase, EfficientSegNet employs MobileNetV3-large as the backbone, leveraging deformable convolutions to enhance channel attention and integrating a lightweight inverted residual block to improve feature reuse, thereby increasing the model’s representational capacity without significantly raising computational cost. For an input image of size H × W, the backbone extracts feature maps at three scales: Branch 1 (H/4 × W/4) captures low-level features containing edges and textures; Branch 2 (H/8 × W/8) contains richer structural information; and Branch 3 (H/16 × W/16) aggregates global semantic information. After extracting high-level features, the CADF module is introduced to further enhance feature representation, generating Branch 4.

During the feature fusion process, Branch 2 is first processed with pointwise convolution to reduce channel dimensions and then fused with Branch 1 using the DWF module to avoid scale mismatches while preserving edge details, resulting in Branch 5 (S_5_ = DWF(Conv_1×1_(S_2_), S_1_)). Similarly, Branch 3 undergoes pointwise convolution and is fused with Branch 2 via the DWF module to strengthen the combination of high-level semantic and mid-level structural features, yielding Branch 6 (S_6_ = DWF(Conv_1×1_(S_3_), S_2_)).

Subsequently, Branch 4 is processed with pointwise convolution to reduce channels and upsampled 2× to increase resolution before being concatenated with Branch 6 to form Branch 7 (S_7_ = Concat(Conv_1×1_(S_4_)_↑2, S_6_)), enriching high-level features with finer details. Further, Branch 7 is compressed with pointwise convolution and upsampled 2× before being concatenated with Branch 5 to form Branch 8 (S_8_ = Concat(Conv_1×1_(S_7_)_↑2, S_5_)), ensuring a comprehensive fusion of semantic and detailed information for clearer boundaries.

Finally, Branch 8 is processed with a 3 × 3 convolution to enhance feature expression and maintain spatial consistency, followed by a 4 upsampling operation that restores the segmentation result to the original input size, yielding the final semantic segmentation output (Sout = (Conv_3×3_(S_8_)_↑4)). This approach, through effective feature extraction, enhancement, and multi-scale fusion, improves segmentation accuracy without compromising computational efficiency, thereby providing an efficient solution for real-time semantic segmentation tasks.

### 3.2. Cascade Attention Dense Field

The Atrous Spatial Pyramid Pooling module captures multi-scale contextual features and extends the receptive field; however, its fixed atrous convolution configuration is unable to adaptively balance global context with local details and often introduces excessive redundant information during feature fusion, which impairs the expression of critical features. Convolutions with large dilation rates capture extensive context but may overlook fine details, whereas those with small dilation rates preserve high spatial resolution for detail capture yet lack sufficient global perception. Thus, balancing global information extraction with detail preservation remains a critical challenge.

To address this issue, we propose the CADF module that fully utilizes the complementarity between features of different scales to achieve effective multi-scale information fusion.

**Figure 2:**
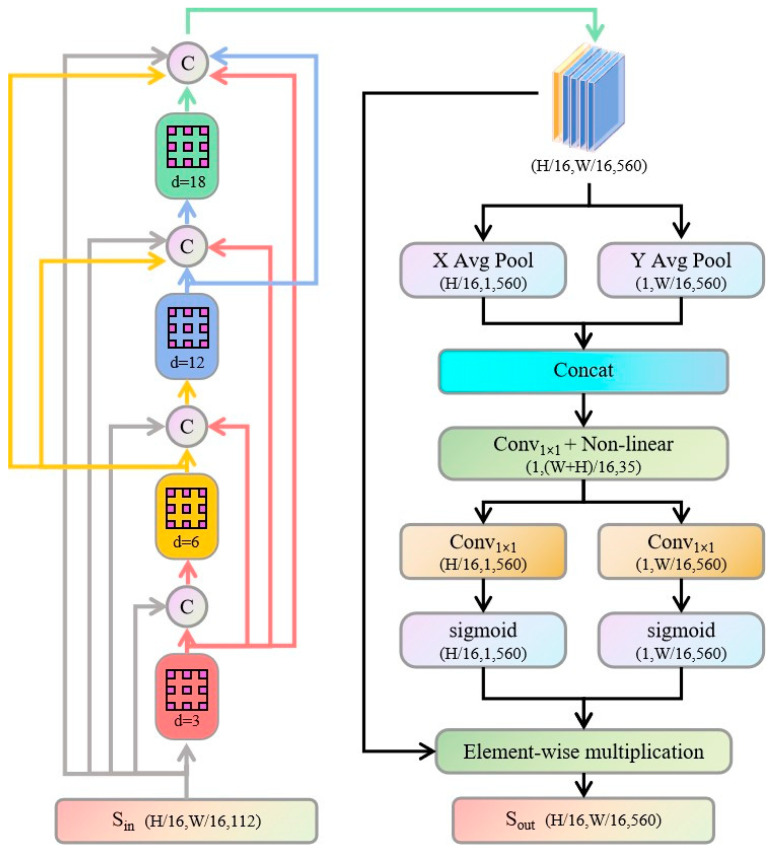
Schematic of CADF module structure.

As shown in Figure 2, the CADF module first extracts multi-scale features from the input feature map Sin using four groups of dilated depthwise separable convolution with dilation rates *r*∈{3, 6, 12, 18}, thereby generating corresponding feature maps *F_r_*:

(1) $$F_{r}=f_{reconfig}(S_{in},r),r\in \left.\left\{3,6,12,18\right.\right\}$$

After obtaining the multi-scale feature maps, these features are concatenated along the channel dimension. To address the redundancy and insufficient multi-scale fusion in the concatenated feature *F_cat_*, we perform global average pooling along both the horizontal and vertical directions to extract long-range dependencies and positional information, thereby enabling the model to better understand contextual relationships in all directions.

(2) $$f_{h}(c,x)=\frac{1}{W}\sum_{i=1}^{W}F_{cat}(c,x,i),x=1,\ldots ,H$$

(3) $$f_{w}(c,y)=\frac{1}{H}\sum_{i=1}^{H}F_{cat}(c,j,y),y=1,\ldots ,W$$

This process produces vectors *f_h_*∈*R^5C×H^* and *f_w_*∈*R^5C×W^* representing horizontal and vertical information, respectively, which are concatenated along the spatial dimension to form a joint descriptive feature.

(4) $$F_{joint}=Concat([f_{h},f_{w}],\dim=-1)\in R^{5C\times (H+W)}$$

A subsequent pointwise convolution followed by a nonlinear activation function transforms this feature into an intermediate representation *F_mid_*,

(5) $$F_{mid}=\delta (Conv_{1\times 1}(F_{joint}))+b_{conv})\in R^{5C^{\prime }\times (H+W)}$$

where *b_conv_* and *δ* denote the bias and activation function, respectively, and *C’* is the number of channels after transformation; this step integrates and compresses information from different directions to provide a compact representation for generating fine attention.

*F_mid_* is then processed through two independent pointwise convolution layers to produce horizontal and vertical attention maps.

(6) $$A_{h}=\sigma (Conv_{1\times 1}(F_{mid})+b_{h})\in R^{5C\times H\times 1}$$

(7) $$A_{w}=\sigma (Conv_{1\times 1}(F_{mid})+b_{w})\in R^{5C\times 1\times W}$$

with *b_h_* and *b_w_* as their respective biases and σ representing the Sigmoid activation function to normalize attention values to the [0, 1] range.

Finally, these attention weights are applied to the horizontal and vertical components of *F_cat_* via element-wise multiplication, achieving dynamic recalibration.

(8) $$S_{out}=F_{cat}⊙A_{h}⊙A_{w}$$

This module not only preserves the advantages of multi-scale features but also effectively suppresses redundant information, thereby significantly enhancing the model’s ability to capture key regions and fine details in complex scenarios, ultimately providing more precise feature support for subsequent segmentation tasks and improving overall segmentation performance.

### 3.3. Dynamic Weighting Feature Fusion

In complex segmentation tasks, deep and shallow features exhibit inherent complementarity. Deep features encode rich semantic information, providing strong support for object recognition; however, their low resolution often leads to the loss of fine details. In contrast, shallow features preserve precise spatial information but struggle to capture sufficient global semantics. Simply adding or concatenating these features may fail to fully leverage their respective strengths and may even introduce redundant information.

To overcome this limitation, we propose the DWF module for fusing low-level and high-level semantic information. Its design employs dynamic weighting and fine-grained embedding to preserve the smoothness and naturalness of high-resolution images while fully integrating deep semantic features, thereby enhancing the representation of target features.

**Figure 3:**
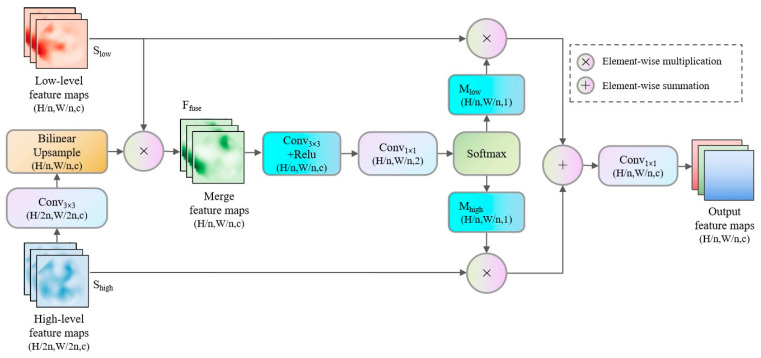
Schematic of DWF module structure.

As depicted in Figure 3, the DWF module first processes the high-level feature *S_high_* with a convolution to obtain enhanced local features *F_high_*. These features are then upsampled via bilinear interpolation to yield continuous, smooth high-resolution features *F_up_*, which compensate for the spatial resolution loss in deep features and better transmit global semantic information at the detail level. Subsequently, *F_up_* is fused with the shallow feature *S_low_*, which retains complete spatial information, through element-wise multiplication to form the preliminary fused feature *F_fuse_* (where ⊗ denotes element-wise multiplication).

(9) $$F_{fuse}=S_{low}⊗(BilinearUp(Conv_{3\times 3}(S_{high})))$$

This direct pixel-wise fusion effectively combines the detailed information of shallow features with the semantic information of the upsampled deep features, laying the foundation for subsequent dynamic weighting.

However, simple fusion can introduce redundancy and fail to reconcile differences between the two feature types. To address this, the DWF module further processes F_fuse_ with a convolution followed by ReLU activation to obtain an intermediate feature *F_nl_*.

(10) $$F_{nl}=RELU(Conv_{3\times 3}(F_{fuse}))$$

Next, two dynamic weight masks, *M_low_* and *M_high_*, are generated via pointwise convolution combined with a *softmax* function.

(11) $$\left[M_{low},M_{high}]=softmax(Conv_{1\times 1}(F_{nl})\right)$$

These masks are applied to the shallow and upsampled deep features, respectively, to achieve refined dynamic fusion.

(12) $$S_{out}=Conv_{1\times 1}(S_{low}⊗M_{low}+F_{up}⊗M_{high})$$

This dynamic weighting mechanism establishes a precise pixel-wise correspondence between global semantic information and local details, suppressing redundancy while enhancing shared semantics, thereby ensuring that key features are prominently expressed in the final output.

Finally, a pointwise convolution adjusts the fused features to match the input dimensions, ensuring the continuity of the network structure and the integrity of information transmission.

## 4. Experiments

### 4.1. Experimental Equipment

**Figure 4:**
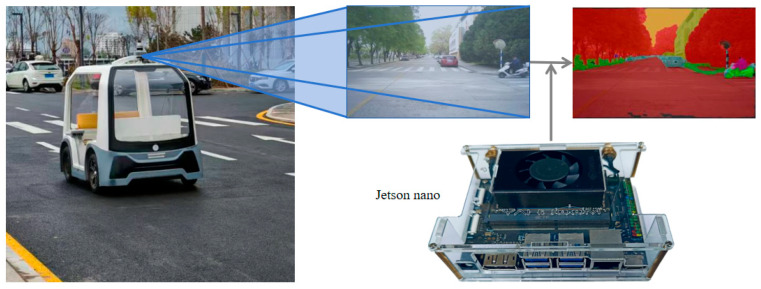
Embedded acquisition system for autonomous driving.

The embedded autonomous driving data acquisition system is shown in Figure 4. This system is built on the Yunle Intelligent Commuter Vehicle, with dimensions of 2179 mm (L) × 1300 mm (W) × 1529 mm (H) and a battery range of 60 km. It supports secondary development and autonomous driving. The video acquisition device used is the Orbbec (LeTV) Astra+, which is mounted at the front of the vehicle. For data processing, the system is equipped with a Jetson Nano mobile development board running the Ubuntu operating system, featuring an Arm Cortex-A78AE processor, an NVIDIA Ampere GPU, and 4 GB of memory.

### 4.2. Experimental Environment

The experiments in this study were implemented using Python 3.7, and all models were trained on an Ubuntu system. The hardware setup includes Intel Core i7-12850HX CPU and NVIDIA RTX A3000 GPU. The software environment consists of PyTorch 1.13.1, CUDA 11.6, and cuDNN 8.5.0.96. The key parameter settings include an image size of 512 × 512 pixels, the Adam adaptive learning rate optimization algorithm with an initial learning rate of 5 × 10^−4^, and a total of 100 training epochs.

### 4.3. Datasets

This study employs three publicly available datasets for comparative experiments: PASCAL VOC 2012 Augmented Dataset [50], PASCAL Context [51], and CityScapes [52].

PASCAL VOC 2012 Augmented Dataset consists of 1449 validation images, 10,582 training images, and 1456 test images, covering 20 semantic categories, including humans, common animals, vehicles, and furniture.

PASCAL Context provides detailed pixel-level annotations for 459 classes, with 4998 training images and 5105 validation images. This dataset includes both indoor and outdoor scenes, but in this study, only 15 commonly used indoor semantic categories are considered.

CityScapes, one of the most authoritative and professional image semantic segmentation benchmarks in autonomous driving, captures street scenes from 50 different cities across various seasons. It contains 2975 training images, 500 validation images, and 1525 test images, categorized into 31 semantic classes. However, this study only utilizes 19 commonly used categories.

### 4.4. Evaluation Metrics

To quantitatively assess the performance of the multi-class semantic segmentation models, this study adopts standard evaluation metrics, defined as follows:

(13) $$mIoU=\frac{1}{k}\sum_{k=1}^{k}\frac{TP_{k}}{TP_{k}+FP_{k}+FN_{k}}$$

(14) $$MPA=\frac{1}{k}\sum_{k=1}^{k}\frac{TP_{k}+TN_{k}}{TP_{k}+FP_{k}+FN_{k}+TN_{k}}$$

The performance evaluation of image segmentation models is based on a confusion matrix comprising four key components: True Positives (*TP*) denote the number of target class pixels correctly identified, reflecting the model’s ability to capture core features; True Negatives (*TN*) represent the number of non-target class pixels accurately excluded, indicating the model’s effectiveness in suppressing background noise; False Positives (*FP*) indicate the number of non-target pixels mistakenly classified as target pixels, which measures the false alarm rate; and False Negatives (*FN*) refer to the number of target pixels incorrectly omitted, reflecting the risk of missed detections.

Based on these definitions, this study employs Mean Intersection over Union (*mIoU*) and Mean Pixel Accuracy (*MPA*) to construct a comprehensive evaluation system. *mIoU* is computed as the mean ratio of the intersection to the union of predicted and ground truth regions across k classes, focusing on quantifying localization accuracy in challenging boundary cases—such as blurred edges and small object segmentation. In contrast, *MPA* averages the global pixel classification accuracy, thereby assessing the model’s overall robustness across different classes. Together, these metrics complement each other: *mIoU* highlights variations in segmentation quality among classes, while *MPA* verifies the model’s stability under imbalanced class distributions, ensuring a thorough and reliable performance evaluation.

## 5. Results

### 5.1. Ablation Experiment

The effectiveness of the multi-scale convolution within the CADF module and its impact on segmentation performance were investigated through multiple ablation experiments. This experiment did not consider depthwise separable convolution and Attention but primarily focused on the contribution of different combinations of dilation rates.

As shown in Table 1, the dilation rate combination of (3, 6, 12, 18) achieved the best performance across all metrics. This configuration preserves global contextual information while retaining local detail, thereby enhancing the model’s precision in delineating object boundaries. Specifically, a dilation rate of 3 aids in capturing small objects and edge textures, bolstering the model’s ability to represent fine structures; dilation rates of 6 and 12 provide medium-scale contextual information that completes the feature representation of target regions; and a dilation rate of 18 effectively covers a wider range of global information, improving semantic consistency in complex scenes. In contrast, the combination of (6, 12, 18) models a limited range of global information but lacks a branch for small dilation rates, weakening its capacity to delineate fine details and resulting in poor edge segmentation. Similarly, while (6, 12, 18, 24) enhances long-range dependency modeling by incorporating a larger dilation rate, the excessive receptive field leads to a loss of local details, thereby reducing segmentation accuracy for small objects and boundary areas. Moreover, although the (3, 6, 12, 18, 24) combination contains richer scale information, the additional branch with a dilation rate of 24 introduces redundant information, ultimately failing to improve segmentation performance and even diminishing the model’s efficiency.

To further investigate the joint influence of convolution types and attention mechanisms in CADF modules, we conducted ablation experiments with four different structural combinations: dilated convolution only (D-Conv), dilated depthwise separable convolution only (DDW Conv), dilated convolution combined with attention (D-Conv + attention), and dilated depthwise separable convolution combined with attention (DDW Conv + attention). From the overall results shown in Table 2, The segmentation accuracy of DDW Conv is slightly higher than that of D-Conv, and the parameter count decreases by 0.81 M. After further introducing the CA attention mechanism, the performance of both types of convolutional structures has been improved. DDW Conv + attention yields the best results, achieving a synergistic optimization of global context awareness and local feature refinement, while maintaining model efficiency. It is the optimal structural design choice in CADF module.

The results presented in Table 3 validate the effectiveness of the DWF module in the feature fusion process. In the model marked as ‘without transition layer’, the decoder directly concatenates and fuses shallow features (H/4 × W/4) from MobileNetV3 with deep features (H/16 × W/16) from ASPP, without employing any mechanism to reconcile the distributional differences between deep and shallow features, leading to semantic misalignment. With the introduction of a transition layer (H/8 × W/8), model performance is notably improved, indicating that transition-based supervision can mitigate multi-scale semantic mismatches. However, direct concatenation-based fusion tends to introduce substantial redundant information, which is not conducive to the lightweight deployment and real-time performance of the model. The DWF module addresses this issue by assigning adaptive weights to feature channels through a dynamic weighting mechanism, thereby reducing redundancy and enhancing the representation of critical information. This facilitates a synergistic optimization of segmentation accuracy and computational efficiency.

### 5.2. Module Ablation Experiment

The effectiveness of the proposed modules was rigorously validated through ablation experiments on the core components of EfficientSegNet, as presented in Table 4. We selected the classic multi-branch parallel structure DeepLabV3 as the baseline model, which achieved a mIoU ranging from 68.09% to 76.95%, serving as the reference for subsequent improvements.

Initially, MobileNetV3 was integrated into the backbone to reduce the model’s parameters and computational complexity. The experimental results indicated that although the parameter count decreased from 54.71 M to 4.84 M, the segmentation performance only experienced a slight drop. This demonstrates that the modified backbone effectively maintains segmentation capability while ensuring computational efficiency, thereby laying the groundwork for a lightweight design.

On the basis of lightweight backbone network, we conducted quantitative analysis on CADF module and DWF module, respectively. The experimental results show that when only the CADF module is introduced, although the computational burden caused by dense connections is alleviated by reducing the number of convolution channels and DDW Conv, the decoder still uses simple concatenation of large channels, which significantly increases FLOPs and parameters, and brings about a significant improvement in mIoU. When only the DWF module is introduced, compared to Baseline+V3, the parameter count is reduced by about 38%, and a moderate improvement in mIoU is achieved. This is because the DWF module first maps high-level channels to low-level channels during feature fusion, and then performs adaptive weighting, effectively avoiding the dilation caused by channel concatenation and significantly reducing the computational complexity of subsequent convolutions.

When the two are combined to build the complete EfficientSegNet, CADF replaces the original ASPP module, which had a high computational cost, significantly reducing the computational load through depthwise separable convolution and compressing the number of convolution channels from 256 to 112. Meanwhile, DWF avoids the computational overhead caused by concatenating high channels in the decoder, ensuring that subsequent convolutions are always performed with low channel counts. The complete model thus integrates the complementary lightweight advantages of DDW Conv and low channel counts, resulting in a further 0.49% reduction in overall computational complexity compared to Baseline+V3+DWF. Compared with the baseline, it not only outperforms all individually improved variants in accuracy but also achieves a substantial improvement of 4.8–5.9% in mIoU, while reducing the number of parameters by 94.6% and FLOPs by 96.9%. These results fully validate that EfficientSegNet can achieve efficient and accurate semantic segmentation while maintaining a lightweight structure.

**Figure 5:**
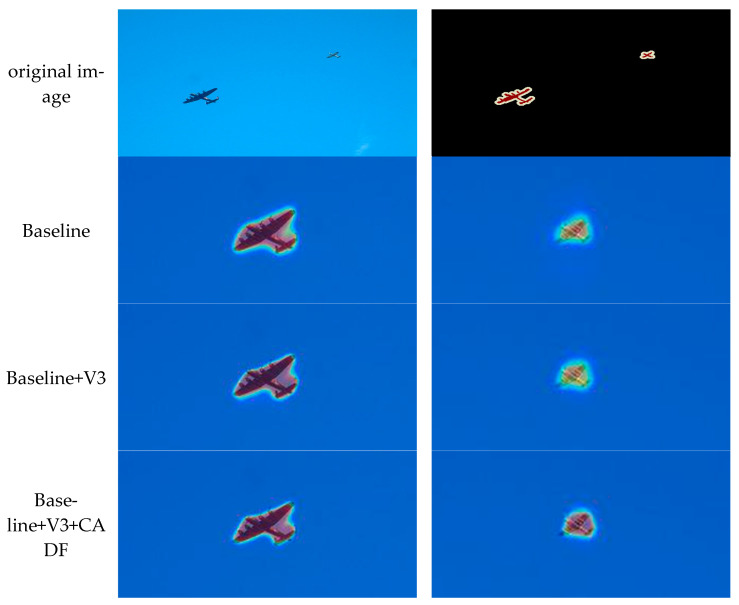

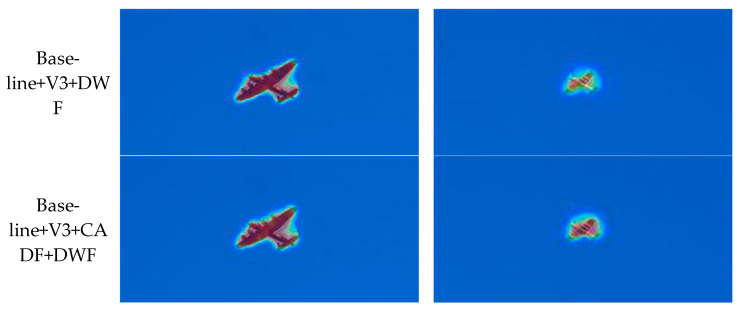
Module ablation experiment attention maps.

In order to more intuitively verify the impact of each optimization module on model performance, Figure 5 presents the attention maps under different configurations. Since the differences in attention are mainly concentrated in small target areas, we enlarged and highlighted the corresponding regions. The results show that after introducing the CADF module, the model exhibits stronger responses to small-scale targets, indicating that the module can effectively integrate multi-scale features and improve the recognition of small objects. With the introduction of the DWF module, the attention shifts more toward the target itself rather than the background or edge regions, demonstrating that its dynamic weighting mechanism can suppress redundant information and highlight key regions during feature selection. When the two modules are combined, the model shows significant improvements in suppressing background noise and focusing on the target, thereby verifying from a visual perspective the complementary roles of the two modules in enhancing overall performance.

### 5.3. Contrast Experiment

To further validate the effectiveness and generalizability of our model in complex semantic segmentation tasks, experiments were conducted on various datasets and compared with other state-of-the-art models, including U-Net [23], PSPnet [24], DeepLab V3+ [29], SFRSeg [37], BiseNet v3 [38] and Mask2Former [41].

As shown in Table 5, on the PASCAL VOC, CityScapes, and PASCAL Context datasets, the mIoU of EfficientSegNet is 1.47%, 7.21%, and 1.67% lower than that of PSPNet, respectively. However, its parameter count is smaller and its prediction speed is faster. On the PASCAL VOC and CityScapes datasets, its segmentation accuracy is comparable to that of BiseNet v3, with differences of 0.39% and 0.81%, respectively. On the PASCAL Context dataset, however, its segmentation accuracy is 5.18% higher, while also achieving higher inference speed. Compared with the lightweight architecture SFRSeg, EfficientSegNet has slightly higher segmentation accuracy, but with an increase of 1.32 M in parameter count, its real-time performance is slightly inferior. Compared with other models, EfficientSegNet performs well in both segmentation accuracy and efficiency. Overall, EfficientSegNet achieves a good balance between segmentation accuracy and efficiency, demonstrating strong overall advantages.

To more intuitively compare the segmentation performance of various models, a series of experiments were conducted on multiple datasets, focusing on aspects such as single-class multi-scale segmentation capability, multi-class multi-scale segmentation capability, edge clarity, and depth detail preservation.

**Figure 6:**
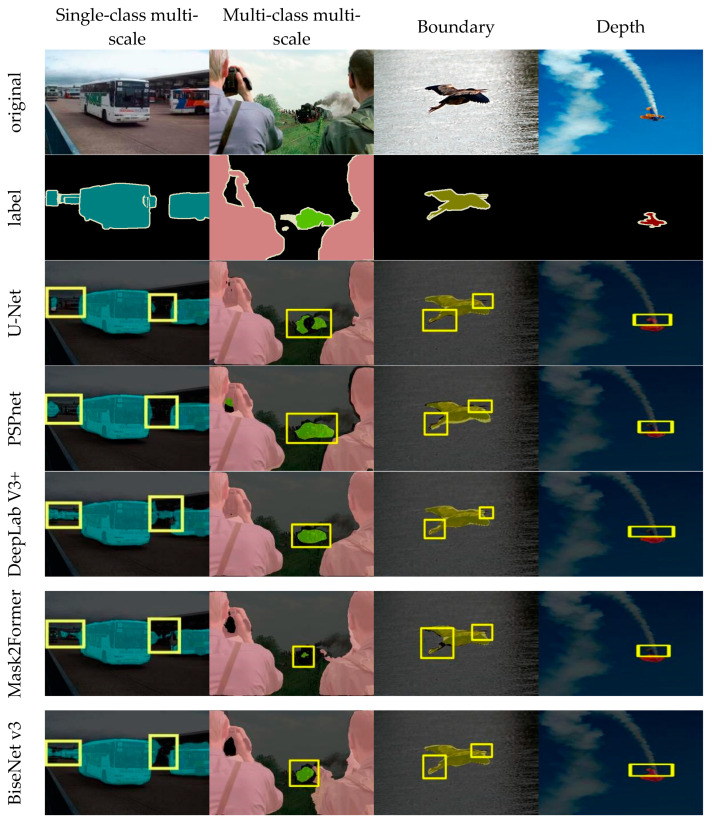

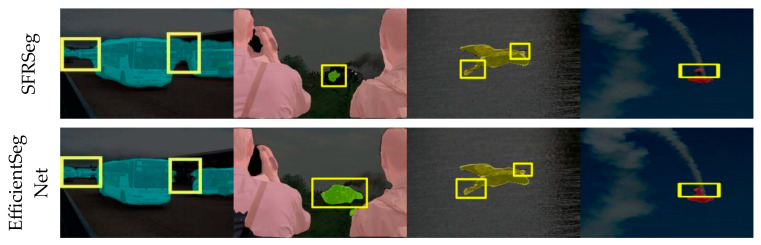
PASCAL VOC visualization results. The yellow box highlights the segmented region of interest.

As shown in Figure 6, in the visualization experiments on the PASCAL VOC dataset, due to the inherent limitations of the encoder–decoder architecture, U-Net and PSPNet performed poorly in handling small and distant targets as well as fine-grained details such as bird beaks, legs, and aircraft tails. DeepLabv3+ has improved semantic consistency in mesoscale scenes through the ASPP module, but errors still occur in large-scale bus regions and aircraft tail fins. Mask2Former emphasizes global context modeling but still falls short in capturing local details, especially in complex structures such as bird legs and aircraft tail fins. Moreover, its global modeling strategy incurs relatively high computational overhead. The shallow semantic representation capacity of BiSeNetV3 is limited, which is mainly reflected in segmentation errors along bus edges. SFRSeg supports fast inference but lacks effective modeling of long-range dependencies, which often leads to semantic inconsistencies within the target, such as disconnections observed in the tail region of an aircraft. In contrast, EfficientSegNet extends the receptive field through CADF modules, effectively preserving global semantic consistency and detailed local structures. For example, it can accurately capture small-scale objects such as buses, trains, and airplane tail fins. Meanwhile, the DWF module adaptively assigns higher weights to boundary-sensitive features, better characterizes the details of complex contours such as bird feet and beaks.

**Figure 7:**
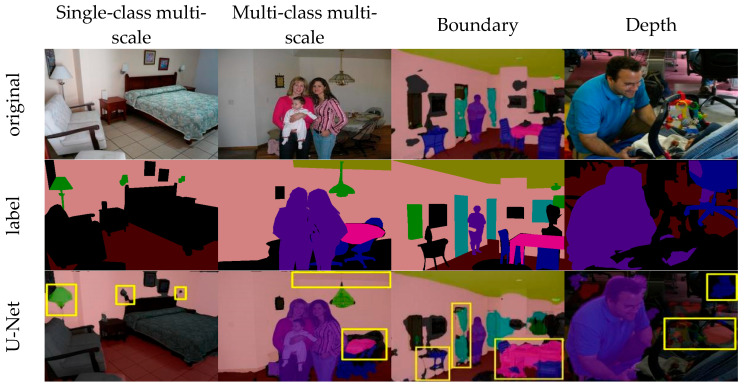

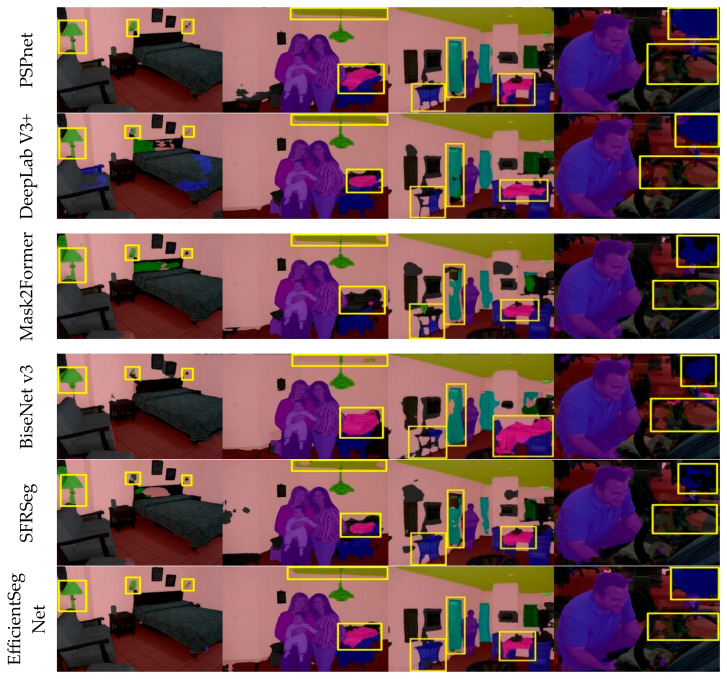
PASCAL Context visualization results. The yellow box highlights the segmented region of interest.

The PASCAL Context dataset is mainly used to evaluate the generalization ability of models in unstructured indoor environments with enclosed spaces, the result is shown in Figure 7. U-Net has difficulty accurately separating corner lights and table legs. PSPNet shows instability when dealing with overlapping regions at wall corners. DeepLabv3+ and SFRSeg exhibit significant semantic discontinuities in structures such as beds and doors. Mask2Former provides better perception of overall structure but still fails to recognize corner lights, table legs, and continuous flooring. BiSeNetV3 lacks wall features in overlapping areas of Multi-class multi-scale scenes. In contrast, EfficientSegNet exhibits strong generalization ability and robustness in complex indoor scenes. The CADF module employs multi-scale dilated convolutions to capture long-range dependencies, enabling the model to maintain semantic consistency between partially occluded and distant targets. This is particularly evident in single-class, multi-scale scenes, where EfficientSegNet preserves contour integrity of lamps even near corners and occluded areas. In addition, the DWF module compensates for missing boundary features in deep networks, facilitating the distinction of highly similar objects such as beds, sofas, tables, and chairs. It also effectively restores connection areas between desktops and table legs, as well as between doors and walls. Moreover, EfficientSegNet demonstrates stronger depth perception and successfully handles complex scenes with cluttered foregrounds, including ground and chairs.

**Figure 8:**
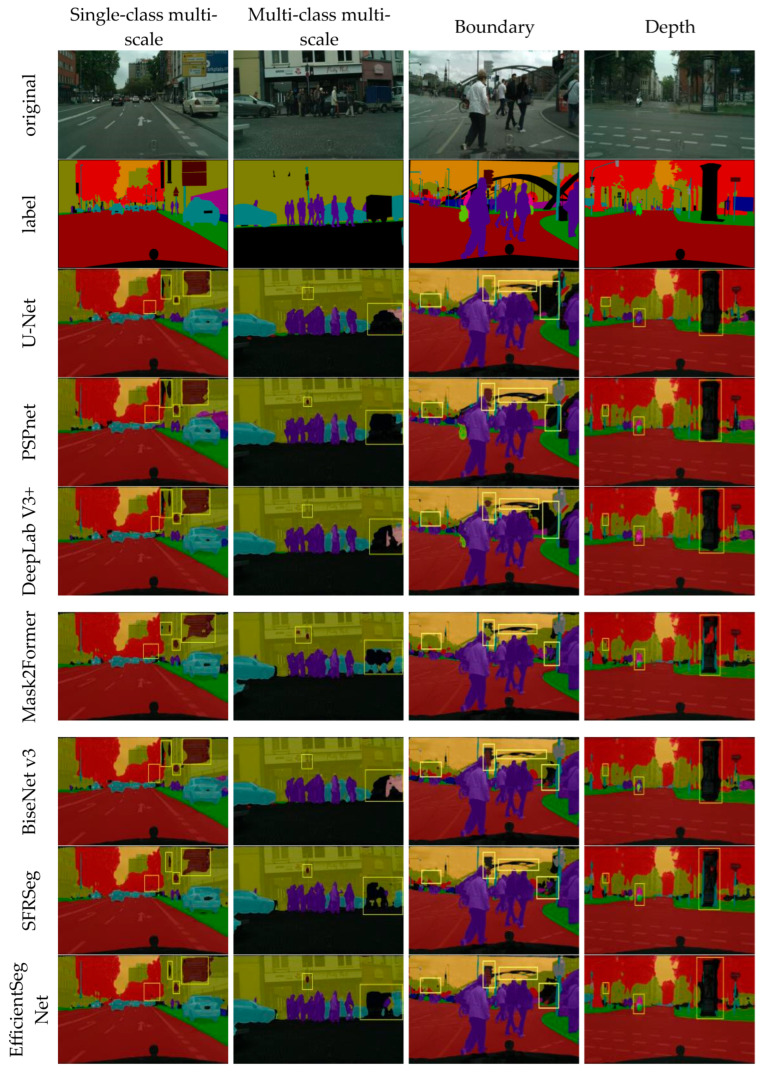
CityScapes visualization results. The yellow box highlights the segmented region of interest.

Figure 8 shows the visualization results of the comparison models on the CityScapes dataset. U-Net is prone to losing small targets such as traffic poles and bicycles in complex scenes. PSPNet improves its global perception ability with the help of the pyramid pooling structure, but it does not provide sufficient features for edge segmentation in areas with dense small targets, such as traffic signs. DeepLabv3+ is insufficient for segmenting the smallest targets, such as bicycle wheels. Mask2Former maintains relatively stable large-scale area segmentation in urban street scenes, but its local feature modeling is weak and cannot accurately segment detailed structures such as traffic signs. The shallow feature representation of BiSeNetV3 is weak, leading to confusion in the segmentation of complex background regions. SFRSeg exhibits structural fragmentation and semantic drift in overlapping areas involving buildings, traffic signals, and other background elements. In contrast, EfficientSegNet shows significant improvement in multi-scale urban scenarios. The CADF module provides clear advantages for multi-scale object recognition, effectively restoring the detailed forms of traffic signs of different sizes while improving the perception of subtle structures such as bicycle wheels and fences. In complex urban scenes with multiple categories, such as pedestrians, vehicles, and buildings, the model maintains stable and detailed segmentation performance, demonstrating strong multi-category adaptability and regional consistency. Furthermore, the DWF module enhances boundary discrimination and optimizes the feature representation of challenging areas such as bridge intersections, traffic signals, and building edges.

In addition, on-site semantic segmentation tests were conducted through the embedded autonomous driving data acquisition system to further validate the model’s generalization ability in real-world application scenarios. The test route started from the South Ring Road of North University of China, driving east to the East Ring Road, then extending north to Central Avenue, then turning west to Xingzhi East Road, and finally returning to the starting point, with a total length of about 850 m.

**Figure 9:**
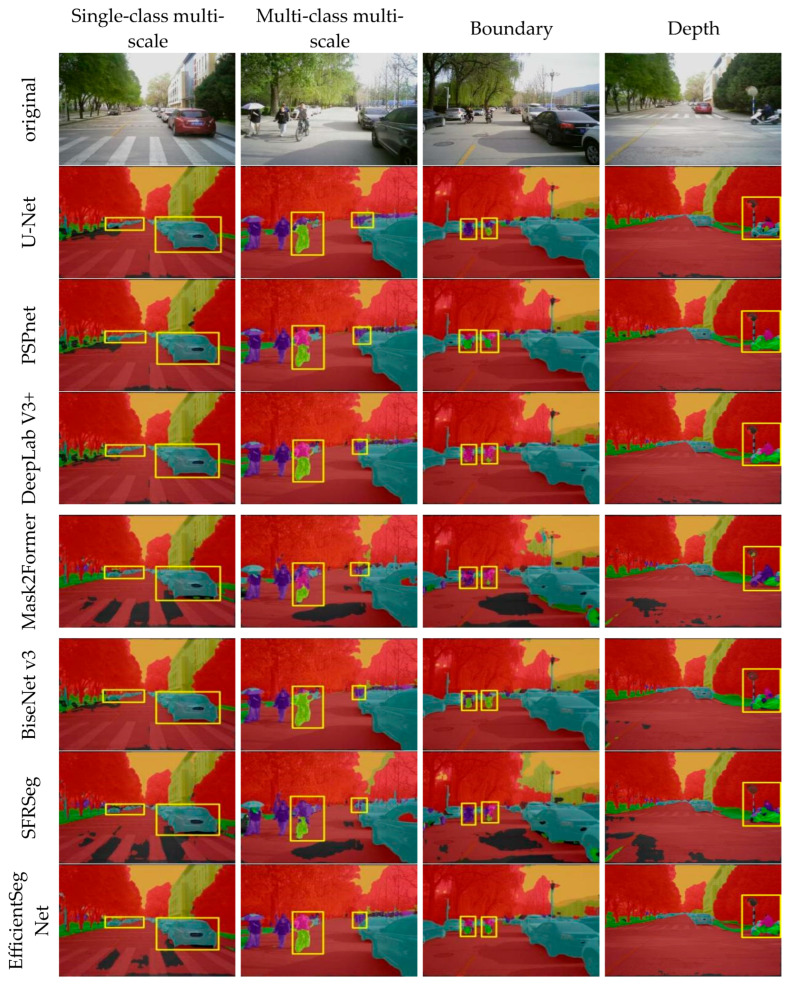
Campus road scenes visualization results. The yellow box highlights the segmented region of interest.

As shown in Figure 9, in the testing of campus road scenes, other models still exhibit issues such as errors, blurred contours, and semantic interruptions. EfficientSegNet, however, produces consistent segmentation results for vehicles of different scales, regardless of whether the objects appear in the center or at the edge of the image. This improvement is largely attributed to the CADF module, which captures cross-scale context and ensures stable semantic consistency between near and distant regions. In scenes containing multiple object categories, such as pedestrians, non-motorized vehicles, and motor vehicles, EfficientSegNet maintains a high degree of category differentiation, even for small-scale targets such as bicycles. The DWF module adaptively emphasizes fine-grained boundary cues and suppresses noise features introduced by shadows and light rays.

Although the collaborative mechanism of CADF and DWF enables EfficientSegNet to achieve robust segmentation performance in scenes with complex interference, there are still issues with incomplete feature extraction and difficulty in focusing on the target object when encountering elongated structures such as fences, streetlights, and table legs, as well as highly overlapping complex backgrounds. In addition, although the model has a certain ability to suppress noise, it is still prone to category confusion in areas with severe lighting changes (such as the intersection of sidewalks and roads), which affects the continuity and accuracy of the segmentation results.

## 6. Conclusions

In this paper, we propose EfficientSegNet, a segmentation model that achieves both high precision and a lightweight design. We theoretically and experimentally demonstrate the importance of integrating deep and shallow features for accurate segmentation. Compared to traditional models, EfficientSegNet effectively balances global semantic understanding with local detail recovery.

This study employs a lightweight backbone network to reduce computational overhead while enhancing embedded deployment performance. Additionally, we introduce the CADF module, which dynamically captures multi-scale information using adaptive atrous convolutions and an improved attention mechanism. This design significantly improves the recognition of road signs, obstacles, and edge details in complex environments. Furthermore, the DWF module efficiently integrates deep and shallow features through bilinear interpolation-based upsampling and pixel-wise weighting. This approach mitigates information loss due to downsampling, enabling precise fine-structure characterization while maintaining global semantic consistency.

Experimental results demonstrate that EfficientSegNet not only achieves superior mIoU performance on standard datasets but also significantly outperforms existing models in terms of robustness when processing dynamic scenarios and complex road environments. Ablation studies further confirm the critical role of the CADF and DWF modules in enhancing segmentation performance, particularly in improving edge detection and small-object segmentation, while maintaining computational efficiency. We argue that this balanced approach combining lightweight architecture and high segmentation accuracy has significant theoretical and practical value.

In conclusion, EfficientSegNet provides a novel solution for effectively integrating deep semantic information with fine-grained spatial details under limited computational resources. Future research will focus on exploring more advanced feature representation mechanisms to enhance the model’s ability to capture slender structural targets while further improving its robustness in environments with severe lighting variations and highly dynamic scenes.

## Figures and Tables

**Table 1:** CADF module expansion rate ablation experiment data.

		(6, 12, 18)	(3, 6, 12, 18)	(6, 12, 18, 24)	(3, 6, 12, 18, 24)
Parameter (M)		5.05	5.75	5.75	6.48
FLOPs (G)		76.17	88.85	88.85	101.67
mIoU (%)	PASCAL VOC	76.81	78.6	78.43	78.79
	PASCAL Context	67.14	69.93	69.06	70.26
	CityScapes	66.84	68.6	68.33	68.85
MPA (%)	PASCAL VOC	87.5	88.3	88.28	89.05
	PASCAL Context	78.57	80.34	80.45	80.65
	CityScapes	77.72	78.71	78.5	79.06

**Table 2:** Ablation results of different CADF structural configurations.

		D-Conv	DDW Conv	D-Conv + Attention	DDW Conv + Attention
Parameter (M)		5.75	4.94	5.87	5.07
mIoU (%)	PASCAL VOC	78.6	78.82	80.51	80.73
	PASCAL Context	69.93	69.85	71.77	71.69
	CityScapes	68.6	68.68	70.30	70.38
MPA (%)	PASCAL VOC	88.3	89.07	89.68	90.45
	PASCAL Context	80.34	80.77	81.32	81.75
	CityScapes	78.71	79.12	80.38	80.79

**Table 3:** Ablation results of DWF module structural design.

		Without Transition Layer	Concatenation Transition Layer	DWF
Parameter (M)		4.84	6.2	3.07
mIoU (%)	PASCAL VOC	76.22	77.24	77.74
	PASCAL Context	67.63	69.2	69.5
	CityScapes	66.56	68.32	67.97
MPA (%)	PASCAL VOC	89.34	90.26	89.99
	PASCAL Context	78.52	78.5	79.33
	CityScapes	77.14	76.96	77.71

**Table 4:** Module ablation experiment data.

		Baseline	Baseline+V3	Baseline+V3+CADF	Baseline+V3+DWF	Baseline+V3+CADF+DWF
Parameter (M)		54.71	4.84	5.07	3.07	2.92
FLOPs (G)		167	48.48	87.3	5.72	5.23
mIoU (%)	PASCAL VOC	76.95	76.22	80.73	77.74	81.13
	PASCAL Context	70.71	67.63	71.69	69.5	74.9
	CityScapes	68.09	66.56	70.38	67.97	71.39
mPA (%)	PASCAL VOC	79.95	89.34	90.45	89.99	90.46
	PASCAL Context	80.1	78.52	81.75	79.33	90.2
	CityScapes	76.96	77.14	80.79	77.71	82.04

**Table 5:** Contrast experiment data.

Model	mIoU (%)			Parameter (M)	Average FPS	
	PASCAL VOC	CityScapes	PASCAL Context		GPU	Jetson Nano
U-Net	67.53	60.6	56.59	43.93	23.33	5.55
PSPnet	82.6	78.6	76.57	46.71	31.68	4.86
DeepLab V3+	76.95	68.09	70.71	54.71	11.3	2.58
Mask2Former	74.1	62.1	59.7	43.96	33.91	1.2
BiseNet v3	80.74	72.2	69.72	39.37	56.49	10.51
SFRSeg	79.3	70.6	61.4	1.6	194	41.1
EfficientSegNet	81.13	71.39	74.9	2.92	76.56	31.88

## Data Availability

The public sources of the data mentioned in this study are described in the paper.
